# Supplementary material for: Feed-forward regulation adaptively evolves via dynamics rather than topology when there is intrinsic noise
Source: Nat Commun. 2019 Jun 3;10:2418. doi: 10.1038/s41467-019-10388-6 (PMC6546794; doi:10.1038/s41467-019-10388-6)
Supplement: Supplementary file 1 — Supplementary Information [file 41467_2019_10388_MOESM1_ESM.pdf]

Supplementary Information

"Feed-forward regulation adaptively evolves via dynamics rather than topology when there is intrinsic noise"

By Xiong et al.

Supplementary Table 1. Major model parameters

Parameter	Values[(1)]	Bounds[(2)]	References
Length of cis-regulatory sequence	150 bp		[1]
Length of TF recognition sequence	8 bp		[2]
Length occupied by a TF on each side of recognition sequence	3 bp		[3]
Dissociation constant between TF and perfect TFBS, $K_d(0)$	**$10^{U(-9,-6)}$ mole per liter**[(3)]	$(0, 10^{-5})$	[4, 5]
Dissociation constant between TF and non-specific DNA, $K_d(3)$	10^{-5} M		[6]
Base rate of transition from Repressed to Intermediate	0.15 min^{-1}		[7]
Maximum transition rate from Repressed to Intermediate	0.92 min^{-1}		[7, 8]
Base rate of transition from Intermediate to Repressed	0.67 min^{-1}		[7]
Maximum transition rate from Intermediate to Repressed	4.11 min^{-1}		Chosen to give same dynamic range and Repressed to Intermediate
Base rate of transition from Intermediate to Active	0.025 min^{-1}		[8]
Maximum transition rate from Intermediate to Active	3.3 min^{-1}		[8]
Transition rate from Active to Intermediate, $r_{Act\_to\_Int}$	**$10^{N(1.27, 0.226)}$ min^{-1}**[(4)]	[0.59, 64.7]	[8-10]
Length of gene, L	**$10^{N(2.568, 0.34)}$ codons**	[50, 5000]	[11, 12]
Rate of transcription initiation, $r_{max\_transc\_init}$	6.75 min^{-1}		[8]
Speed of transcription elongation	600 codon min^{-1}		[13-15]
Time for transcribing UTRs and for terminating transcription	1 min		[13-15]
Rate of mRNA degradation, r_{mRNA_deg}	**$10^{N(-1.49, 0.267)}$ min^{-1}**	$[7.5\times10^{-4}, 0.54]$	[16]
Speed of translation elongation	330 codon min^{-1}		[17]
Translation initiation time	0.5 min		[17]
Protein synthesis rate, $r_{protein\_syn}$	**$10^{N(0.322, 0.416)}$ molecule $mRNA^{-1}$ min^{-1}**	$[4.5\times10^{-3}, 61.4]$	[17]
Rate of protein degradation, $r_{protein\_deg}$	**$10^{N(-1.88, 0.561)}$ min^{-1}**	$[3.0\times10^{-6}, 0.69]$	[18]
Saturation concentration of effector protein, N_{e_sat}	10,000 molecules $cell^{-1}$		[19]
Fitness cost of protein expression for a gene with $L = 10^{2.568}$, c_{transl}	2×10^{-6} $molecules^{-1}$ min^{-1}		[19, 20]
Maximum number of effector gene copies	5		
Maximum number of TF gene copies, excluding the signal	19		

(1) Parameters in bold can be altered by mutation, and the table shows the distributions from which their initial values are sampled. Estimation of N_{e_sat} is described in the Methods; estimation of the other parameters is described in the Supplementary Methods.

(2) Same units as the parameter values. Parentheses mean the parameter cannot take the boundary values; square brackets mean it can. We also use these bounds to constrain mutation (see Supplementary Methods).

(3) The uniform distribution is denoted U(min, max).

(4) The normal distribution is denoted N(mean, SD).

Supplementary Table 2. Mutation rates and effect sizes

Mutation	Relative rate	Effect of mutation[1]
Single nucleotide substitution	5.25×10^{-8} per gene	
Gene deletion	1.5×10^{-7} per gene[2]	
Gene duplication	1.5×10^{-7} per gene[2]	
Mutation to consensus sequence of a TF	3.5×10^{-9} per gene	
Mutation to TF identity (activator vs. repressor)	3.5×10^{-9} per gene	
Mutation to $K_d(0)$	3.5×10^{-9} per gene	k = 0.5, μ = -5[2], σ = 0.776
Mutation to L	1.2×10^{-11} per codon	
Mutation to $r_{protein\_syn}$	9.5×10^{-12} per codon	k = 0.5, μ = 0.021[2], σ = 0.760
Mutation to $r_{protein\_deg}$	9.5×10^{-12} per codon	k = 0.5, μ = -1.88, σ = 0.739
Mutation to $r_{Act\_to\_Int}$	9.5×10^{-12} per codon	k = 0.5, μ = 1.57[2], σ = 0.773
Mutation to r_{mRNA_deg}	9.5×10^{-12} per codon	k = 0.5, μ = -1.19, σ = 0.396

(1) Mutation to these quantitative rates takes the form $\log_{10}x' = \log_{10}x + \mathrm{Normal}(k(\mu - \log_{10}x), \sigma)$, where x is the original value of the rate and x' is the value after mutation. See Supplementary Methods for details.

(2) The value of this parameter is different during burn-in. See Supplementary Methods for details.

Supplementary Table 3. Evolutionary constraint on parameters in AND-gated C1-FFLs

	Signal		TFs		Effector	
	V_n / V_s	M_s / M_n	V_n / V_s	M_s / M_n	V_n / V_s	M_s / M_n
$r_{Act\_to\_Int}$	NA	NA	0.89	0.18	8.26	0.13
r_{mRNA_deg}	NA	NA	2.09	0.98	13.4	2.55
$r_{protein\_syn}$	NA	NA	1.51	8.03	43.1	62.4
$r_{protein\_deg}$	NA	NA	1.28	0.56	7.23	12.5
$K_d(0)$	0.68	0.002	0.67	0.009	NA	NA
Locus length	NA	NA	1.01	0.72	2.07	0.79

Adaptive AND-gated C1-FFLs are taken from the 25 high-fitness replicates evolved for filtering out a spurious signal, where the signal directly regulates the effector. We calculate the variance V_s of each parameter across the replicates. For each replicate, we sample one of the last 10,000 evolutionary steps. If the genotype at the sampled evolutionary step contains no AND-gated C1-FFLs, we resample to replace it with a different evolutionary step. If it contains more than one AND-gated C1-FFL, we sample one motif. We then take the variance V_s of each parameter value across the 25 replicates. We repeat this sampling process 100 times (using the same 25 replicates) and take the mean in order to obtain a better estimator of the variance in each parameter value. We compare this by a comparable variance V_n given no selection. We obtain V_n from 30 evolutionary replicates under no selection (from Fig. 6), sampling parameter values from the signal, from one TF gene copy, and from one effector gene, without the requirement for C1-FFL presence. Variances are calculated for log-transformed parameter values, except for locus length. For locus length, we use the coefficient of variation rather than variance, i.e. we divide each variance by the square of the respective average locus length. We use the ratio V_n / V_s to measure the degree of evolutionary constraint experienced by a parameter. Natural selection is expected to reduce V_s when the functionality of AND-gated C1-FFLs relies on particular values of that parameter. The table also shows how the mean parameter values M_s in adaptive AND-gated C1-FFLs differ from the expected value M_n given no selection. M_s and M_n are calculated as arithmetic means for locus length and as geometric means for all other parameters. Large M_s / M_n indicates that selection tends to increase the value of a parameter. The ratio V_n / V_s is greater than 1 (indicating constraint), for most parameters. $K_d(0)$ has a notably small variance ratio; nevertheless, the ratio of mean $K_d(0)$ values indicates that $K_d(0)$ is subject to strong directional selection. Effectors are more constrained than TFs, likely because the former are less redundant, having evolved fewer gene copies (4.7 on average for effectors vs. 8.6 for TFs). High degradation rates of effector mRNA and protein suggest selection to shorten the impact of transient expression in response to a short spurious signal (Supplementary Figure 3). High degradation rates of effector mRNA and protein are also seen in Supplementary Table 4 and Supplementary Table 5.

Supplementary Table 4. Evolutionary constraint on parameters in AND-gated diamonds

	Signal		Fast TFs		Slow TFs		Effector	
	V_n / V_s	M_s / M_n	V_n / V_s	M_s / M_n	V_n / V_s	M_s / M_n	V_n / V_s	M_s / M_n
$r_{Act\_to\_Int}$	NA	NA	1.49	0.44	1.15	0.18	6.64	0.1
r_{mRNA_deg}	NA	NA	5.27	8.21	1.07	0.81	7.99	2.34
$r_{protein\_syn}$	NA	NA	2.10	16.2	1.09	4.96	139	57.8
$r_{protein\_deg}$	NA	NA	12.5	45.3	1.53	0.99	25.7	11.3
$K_d(0)$	0.65	0.005	0.30	0.004	0.18	0.007	NA	NA
Locus length	NA	NA	3.43	0.47	3.40	0.47	5.97	0.74

V_n, V_s, M_n, and M_s are defined in the same way as in Supplementary Table 3, and are calculated from 18 high-fitness evolutionary replicates (Fig. 7b) in which isolated AND-gated diamonds occur in at least 100 of the last 10,000 evolutionary steps. Because they occur at low rates, we sample 50 times per evolutionary replicate, instead of 100 times as in Supplementary Table 3 and Supplementary Table 5. There is more constraint on fast TFs than on slow TFs. The fast TFs usually have more gene copies than the slow TFs, therefore redundancy is not the reason for this difference in constraint. As seen for the C1-FFLs in Supplementary Table 3, effectors are more constrained than either TF, $K_d(0)$ shows strong selection for high affinity combined with high variance, and effectors evolve rapid degradation. Fast TFs exhibit not just fast protein degradation (which was used for their identification), but also fast mRNA degradation.

Supplementary Table 5. Evolutionary constraint on parameters in isolated AND-gated C1-FFLs

	Signal		Signal-regulated TFs		TF-regulated TFs		Effector	
	V_n / V_s	M_s / M_n	V_n / V_s	M_s / M_n	V_n / V_s	M_s / M_n	V_n / V_s	M_s / M_n
$r_{Act\_to\_Int}$	NA	NA	2.16	0.33	1.03	0.26	6.81	0.13
r_{mRNA_deg}	NA	NA	10.8	8.5	1.40	0.74	12.4	2.36
$r_{protein\_syn}$	NA	NA	4.34	24.9	2.35	9.83	119	58.6
$r_{protein\_deg}$	NA	NA	73.6	49.4	1.50	0.34	34.1	9.92
$K_d(0)$	0.51	0.005	0.29	0.009	0.24	0.002	NA	NA
Locus length	NA	NA	2.52	0.71	2.45	0.71	3.35	0.73

V_n, V_s, M_n, and M_s are defined in the same way as in Supplementary Table 3, and are calculated from 12 high-fitness evolutionary replicates (Fig. 7b) evolved when the signal cannot directly regulate the effector, and in which isolated AND-gated C1-FFLs occur in at least 1,000 out of the last 10,000 evolutionary steps. Note that the signal-regulated TFs, which are identified via network topology, also have high protein degradation rates, as is used to identify their fast TF counterparts in diamonds – they can thus be seen as a kind of fast TF. Consistent with results on C1-FFLs when direct regulation is allowed (Supplementary Table 3) and results on isolated AND-gated diamonds (Supplementary Table 4), effectors are more constrained than signal-regulated (fast) TFs, which are more constrained than TF-regulated (slow) TFs, despite an opposite trend in gene copy number. Note that selection promotes fast mRNA and protein degradation in fast TFs, but promotes slow degradation of slow TFs; this result is also found more weakly in Supplementary Table 4.

Supplementary Table 6. Summary of mutations that replaced the resident genotype

	Probability that mutation of this type is accepted, given it occurs		Probability that an accepted mutation is of this type	
	First 1,000 evol. steps	Last 1,000 evol. steps	First 1,000 evol. steps	Last 1,000 evol. steps
Substitution	0.34 ± 0.01	0.35 ± 0.00	0.180 ± 0.005	0.213 ± 0.008
Deletion	0.27 ± 0.01	0.21 ± 0.01	0.360 ± 0.003	0.345 ± 0.005
Duplication	0.34 ± 0.01	0.32 ± 0.01	0.368 ± 0.003	0.343 ± 0.005
TF recognition seq.	0.30 ± 0.02	0.19 ± 0.02	0.009 ± 0.001	0.005 ± 0.000
$r_{\mathrm{Act\_to\_Int}}$	0.33 ± 0.02	0.25 ± 0.01	0.012 ± 0.001	0.010 ± 0.001
$r_{\mathrm{mRNA\_deg}}$	0.34 ± 0.02	0.27 ± 0.01	0.014 ± 0.001	0.016 ± 0.002
$r_{\mathrm{protein\_syn}}$	0.32 ± 0.02	0.23 ± 0.01	0.013 ± 0.001	0.013 ± 0.001
$r_{\mathrm{protein\_deg}}$	0.35 ± 0.01	0.26 ± 0.01	0.014 ± 0.001	0.015 ± 0.002
$K_{\mathrm{d}}(0)$	0.28 ± 0.02	0.21 ± 0.02	0.006 ± 0.000	0.005 ± 0.001
TF identity	0.29 ± 0.01	0.29 ± 0.02	0.008 ± 0.000	0.008 ± 0.001
Locus length	0.33 ± 0.01	0.36 ± 0.01	0.017 ± 0.001	0.026 ± 0.002

Data is shown as mean $\pm$ s.e.m. over the 45 evolutionary replicates under selection for filtering out a spurious signal, with the signal allowed to regulate the effector directly. Without selection, each mutation would have probability 50% of replacing the resident; selection reduces this to around one in three at the beginning of the simulation, down to around one in four at the end. This high rate of accepting mutations after fitness has plateaued suggests significant nearly neutral evolution, i.e. that slightly deleterious mutations fix and are then compensated for. The estimated selection coefficient need only be 10^{-8} for a mutant to replace the resident, which can easily occur for a slightly deleterious mutation through the error in fitness estimation (see Evolution Simulation in the main text). Single nucleotide substitutions are particularly prone to nearly neutral evolution, whereas changes to the consensus sequence recognized by a TF are under stronger stabilizing selection. Deletion and duplication mutations are the most common forms of substitution not because they are more likely to be accepted, but because they occur at higher mutation rates.

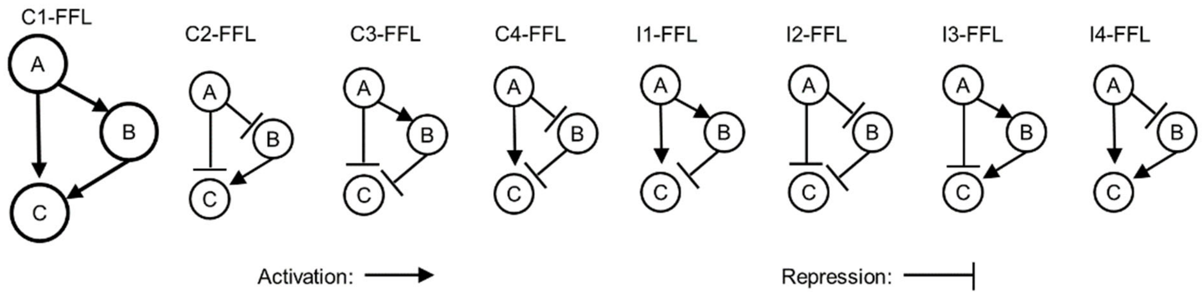

Supplementary Figure 1. Feed-forward loops come in eight subtypes. TF A and TF B can activate (indicated by arrows) or repress (indicated by bars) expression of the effector C as well as other TFs. Auto-regulation is allowed, but not shown. Following Milo et al.[21], we exclude the case in which A and B regulate one another, rather than treating this case as two overlapping FFLs. C stands for coherent and I for incoherent.

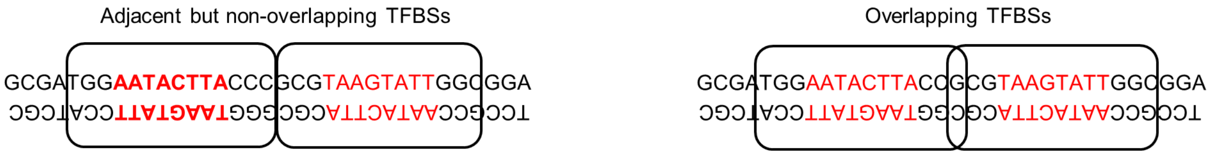

Supplementary Figure 2. Spatial hindrance of TF binding. TFs (white boxes) recognize 8 bp (red) sites while occupying and thus excluding other TFs from a 14 bp long space (see Supplementary Methods for justification of the parameterization). TFs are assumed to bind in either orientations [22]. The sequence on the left allows simultaneous binding but that on the right does not.

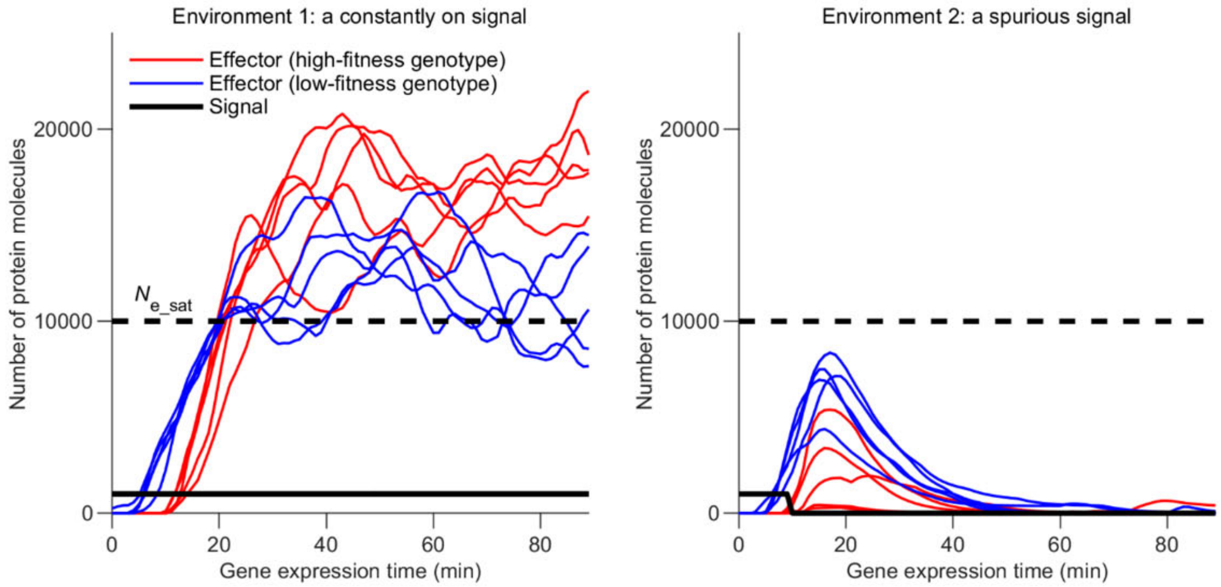

Supplementary Figure 3. Examples of evolved phenotypes under selection for filtering out a short spurious signal. The figure shows trajectories of the effector protein in one randomly chosen high-fitness replicate (red) and one randomly chosen low-fitness replicate (blue), as defined in Fig. 4a. The genotype of the final evolutionary step is used, and other genotypes were confirmed to behave similarly. Each genotype is illustrated by 5 replicate gene expression simulations in each of the two environments. The high-fitness genotype has a longer delay followed by more rapid response given a consistent signal, with this longer delay reducing but not eliminating effector expression given a short spurious signal. The signal is allowed to directly regulate the effector in these simulations. The burn-in period is not shown, with gene expression time zero corresponding to the moment the signal is turned on. Among gene expression replicates of the same genotype, the concentration at a given time usually has an approximately log-normal distribution, but in environment 2 the distribution has two modes after the spurious signal turns off. One mode corresponds to expression at the basal rate, the other to a burst of expression that has yet to turn off. Because of this bimodality, we plot sample trajectories rather than mean concentration over many replicates.

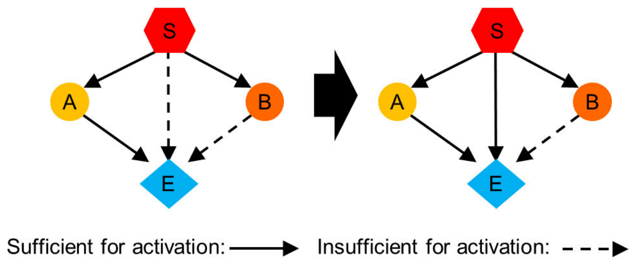

Supplementary Figure 4. Examples of confounding motifs in perturbation analysis. The TRN on the left contains a slow TF-controlled C1-FFL (S-A-E) and an AND-gated C1-FFL (S-B-E). To convert S-B-E into a signal-controlled C1-FFL, we need to add one TFBS for the signal to the cis-regulatory sequence of E. However, this change also makes S-A-E OR-gated, making it difficult to conclude whether it is the AND gate logic of S-B-E that matters for fitness. See Supplementary Methods for other confound TRNs that are excluded from perturbation analysis.

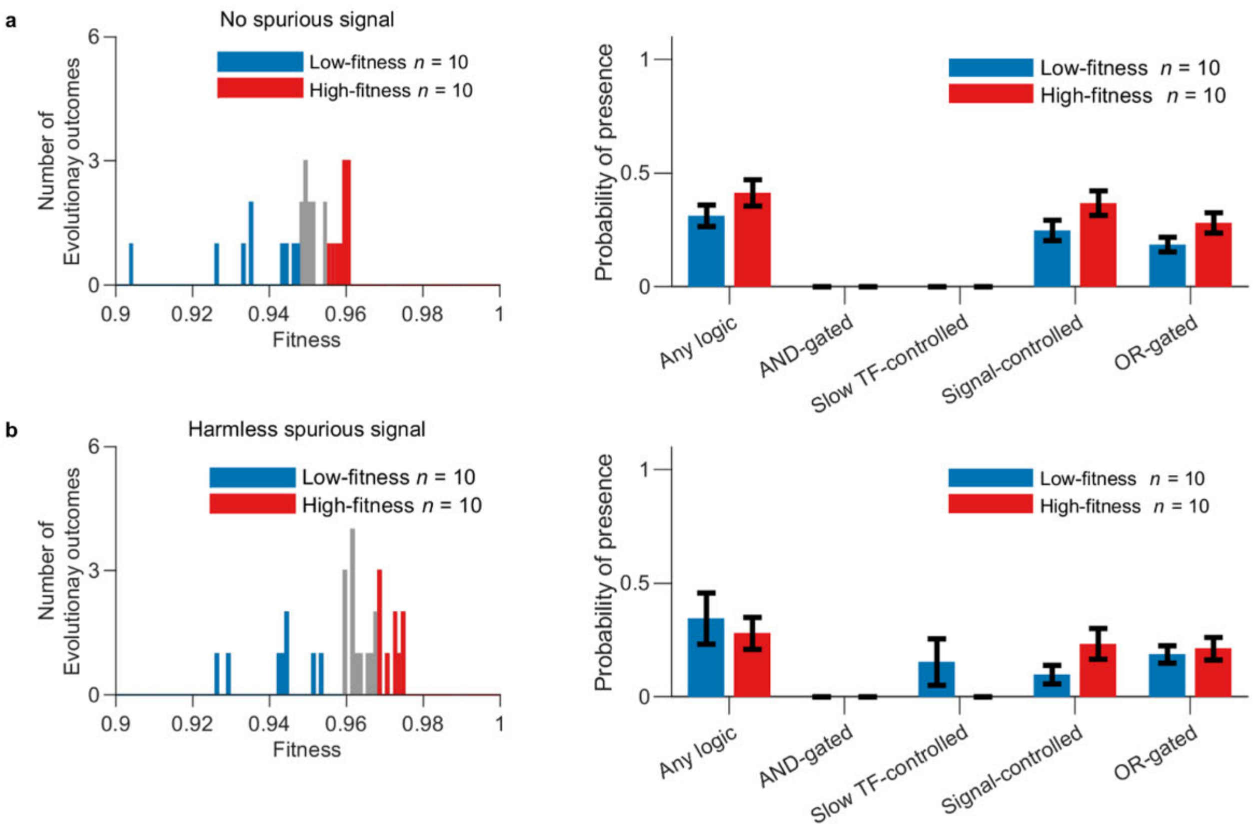

Supplementary Figure 5. Genotypes evolved under control selective conditions. Evolutionary outcomes under **a** no spurious signal, and **b** harmless spurious signal. There is no clear evidence of a multimodal distribution of fitness outcomes among replicates (left), and C1-FFLs occur equally in the 10 genotypes of the highest fitness vs. the 10 genotypes of the lowest fitness (right), and so the entire distribution (left) was used to produce Fig. 6. Data are shown as mean ± s.e.m. over evolutionary replicates.

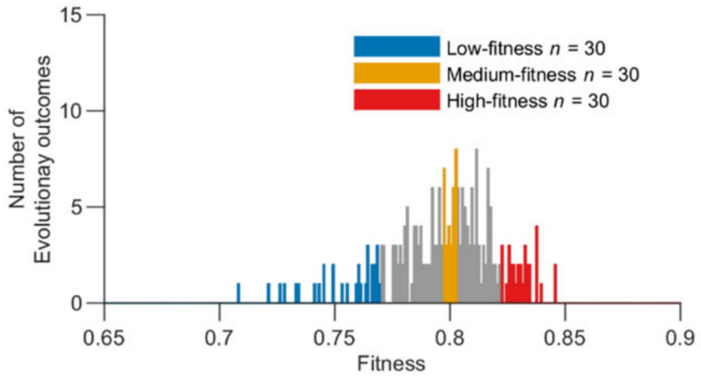

Supplementary Figure 6. Fitness of spurious signal filters under indirect regulation. We performed 238 evolutionary replicates under selection for filtering out short spurious signals, when the signal cannot directly regulate the effector. The fitness of a replicate is the average genotype fitness over the last 10,000 evolutionary steps. Colors indicate replicates analyzed elsewhere.

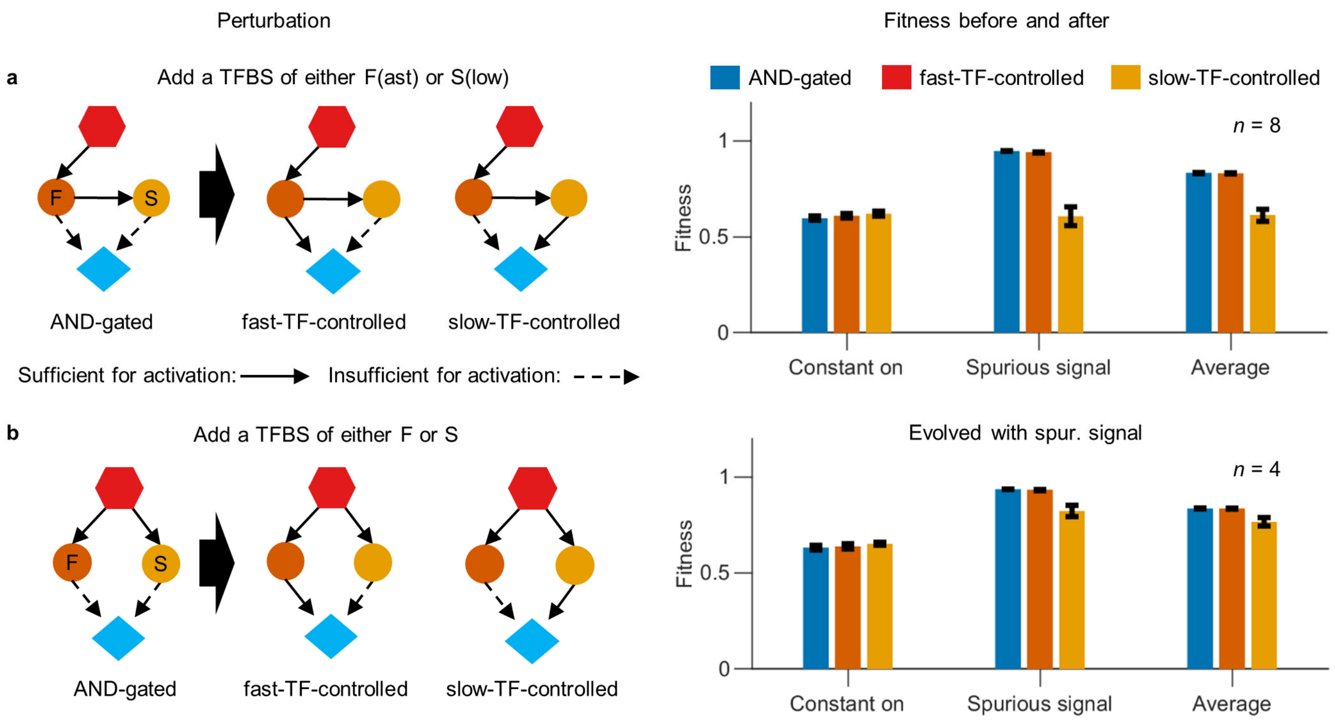

Supplementary Figure 7. Weak TFBSs in the effector are tolerated for the slow but not fast TF**.** We add a 2-mismatch TFBS of either the fast TF or the slow TF to break the AND gate. Allowing the effector to respond to the slow TF alone slightly increases the ability to respond to the signal, but leads to a larger loss of fitness when effector expression is undesirable. Allowing the effector to respond to the fast TF alone does not significantly change fitness. We perform the perturbation on **a** 8 of the 18 high-fitness replicates from Fig. 7b that evolved an AND-gated C1-FFL, and **b** 4 of the 26 high-fitness replicates that evolved an AND-gated diamond in Fig. 7b. Replicate exclusion was based on the co-occurrence of other motifs with the potential to confound results (see Supplementary Methods for details). Fitness is shown as mean ± s.e.m. of over replicate evolutionary simulations, calculated as described for Fig. 5.

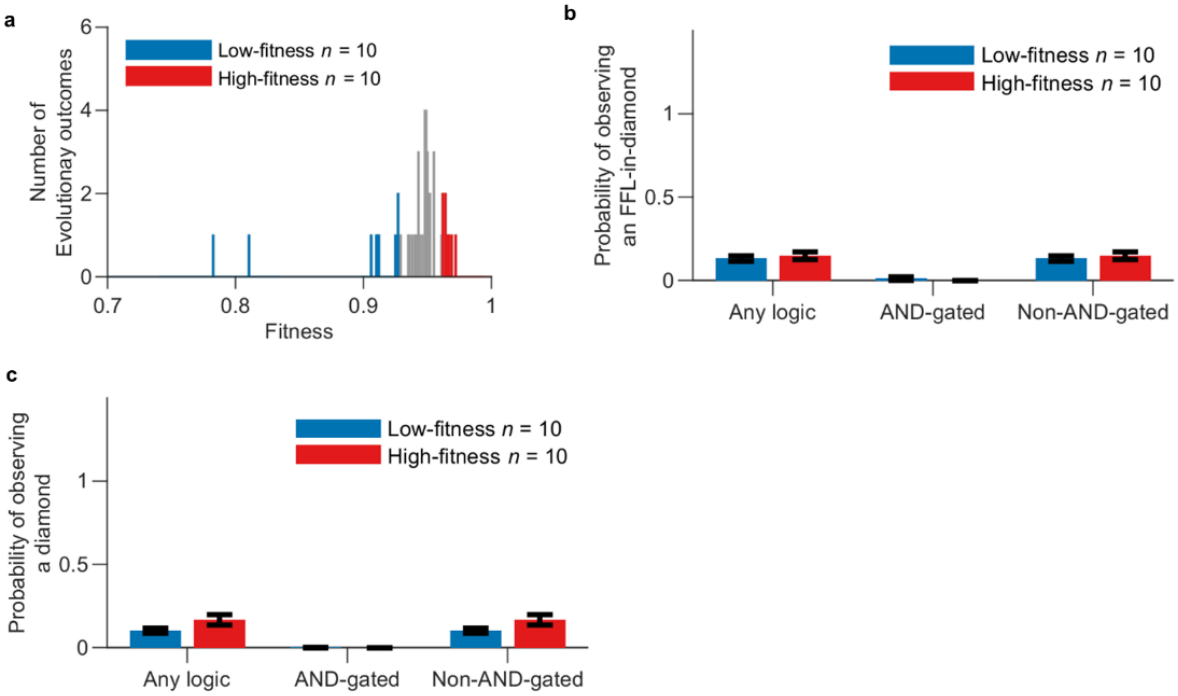

Supplementary Figure 8. Evolution under a harmless spurious signal and indirect regulation. Genotypes are evolved to filter out a harmless spurious signal, when the signal is not allowed to directly regulate the effector. **a** Fitness distribution of 50 replicate simulations. The occurrence of both **b** FFL-in-diamonds and **c** isolated diamonds were similar in the 10 genotypes with the highest fitness vs. in 10 genotypes with the lowest fitness. Weak (two-mismatch) TFBSs are included when scoring motifs. Data are shown as mean ± s.e.m. over replicates. Isolated C1-FFLs rarely evolve under this condition, therefore their occurrence is not plotted.

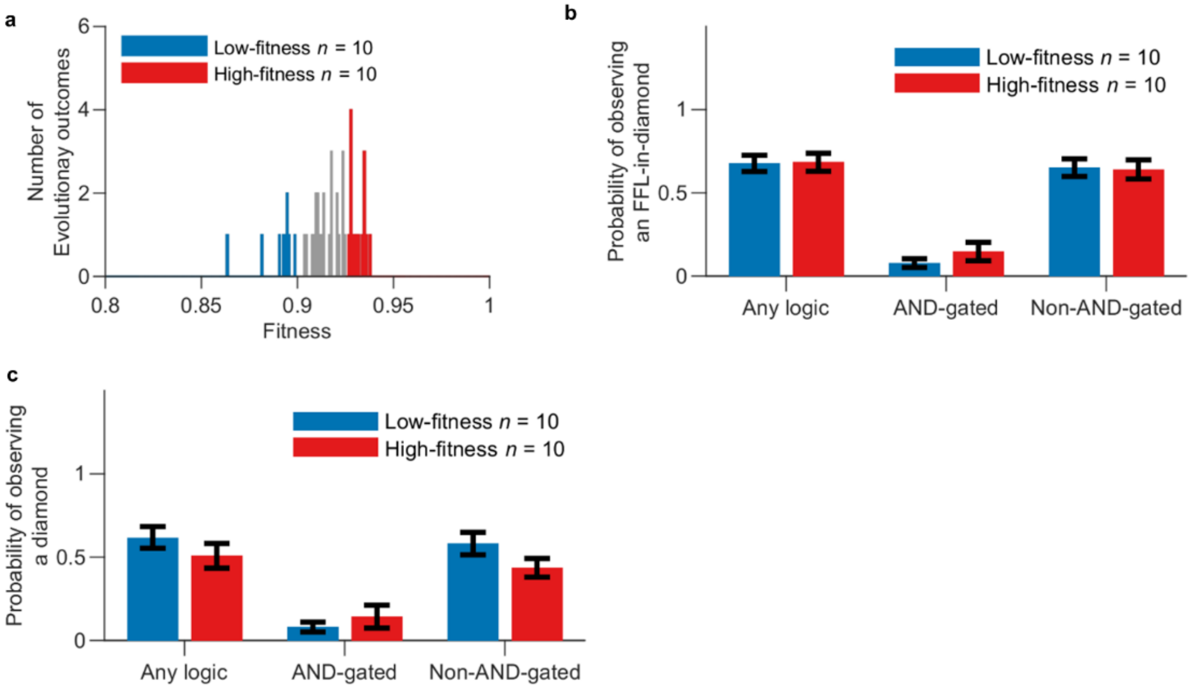

Supplementary Figure 9. Evolution under no spurious signal and indirect regulation. Genotypes are evolved when there is no spurious signal, when the signal is not allowed to directly regulate the effector. **a** Fitness distribution of 46 replicate simulations. The occurrence of both **b** FFL-in-diamonds and **c** isolated diamonds were similar in the 10 genotypes with the highest fitness vs. in the 10 genotypes with the lowest fitness. Weak (two-mismatch) TFBSs are included when scoring motifs. Data are shown as mean ± s.e.m. over replicates. Isolated C1-FFLs rarely evolve under this condition, therefore their occurrence is not plotted.

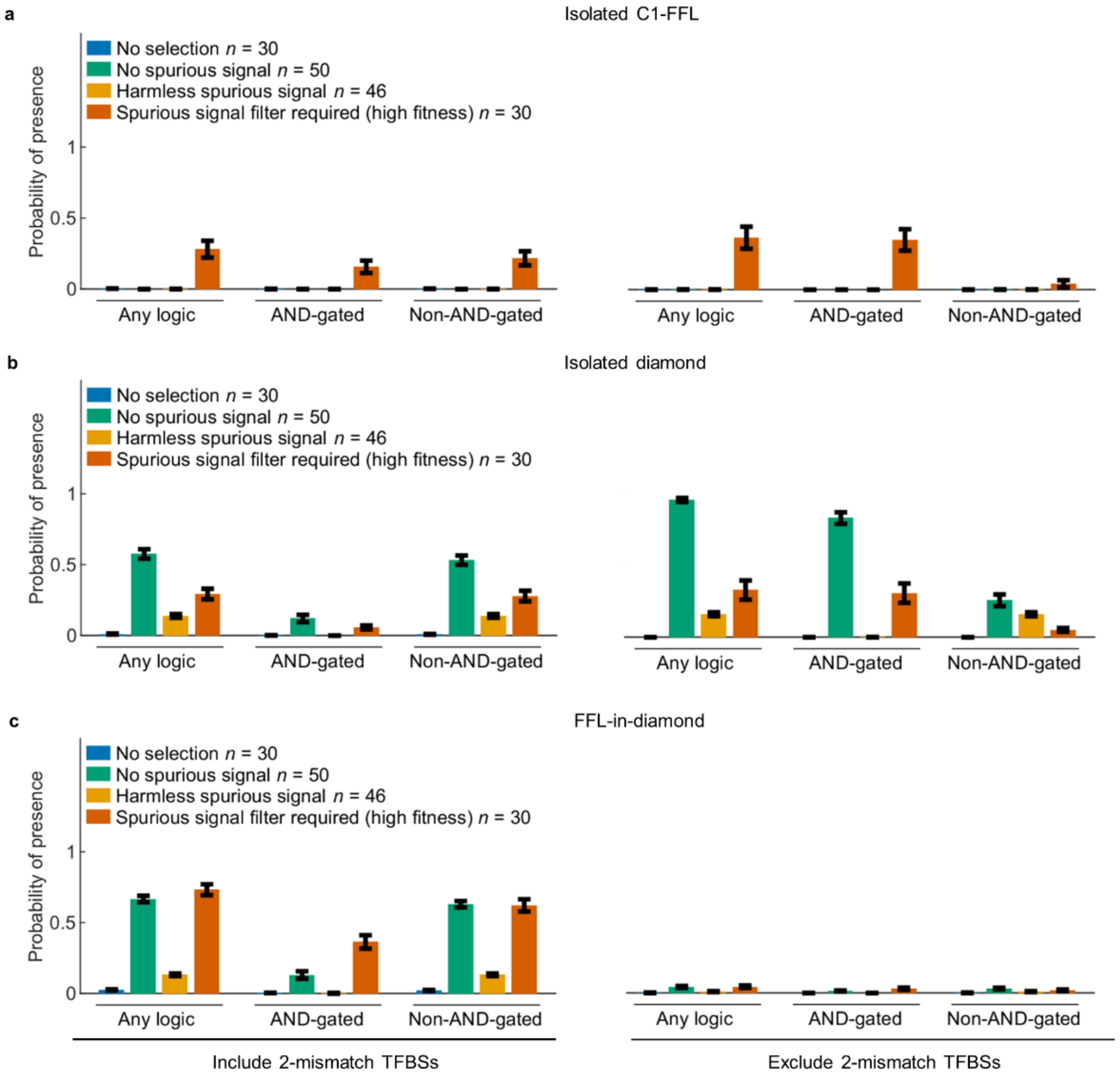

Supplementary Figure 10. AND-gated C1-FFLs and diamonds evolved under different conditions. The signal is not allowed to directly regulate the effector. Frequencies of evolved **a** isolated C1-FFLs, **b** isolated diamonds, and **c** FFL-in-diamonds. While AND-gated isolated C1-FFLs evolved only under selection for filtering out a spurious signal, AND-gated isolated diamonds also evolve in the absence of spurious signals. When scoring motifs, we either include (left) or exclude (right) all two-mismatch TFBSs in the cis-regulatory sequences of intermediate TF genes and effector genes. The right panels of **a** and **b** are identical to Fig. 10. We excluded "no regulation" (Fig. 2) diamonds from the "Any logic" and "Non-AND-gated" tallies in **b**; this was necessary because of their high occurrence due to duplication and divergence of intermediate TFs. See Supplementary Methods for the calculation of the y-axis. Data are shown as mean ± s.e.m. over evolutionary replicates.

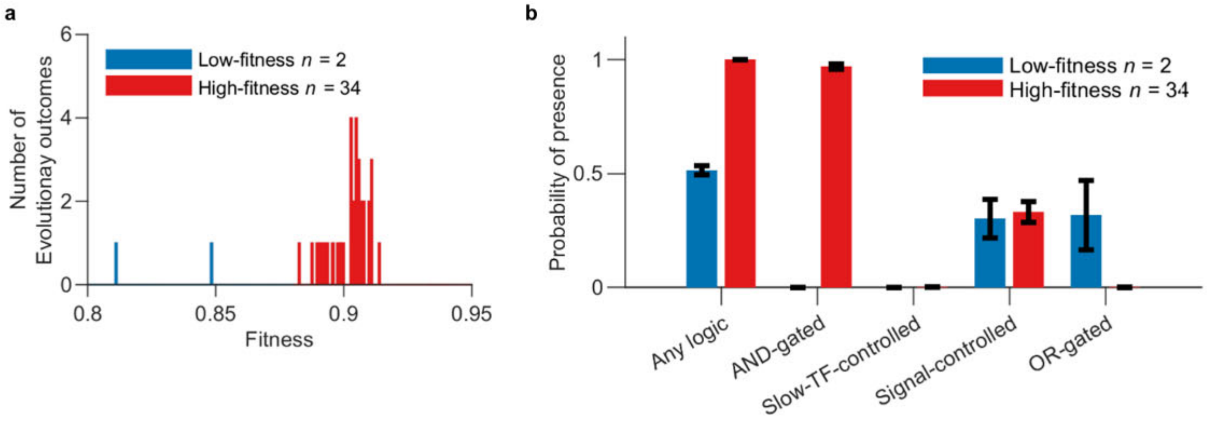

Supplementary Figure 11. After removing cost of gene expression, AND-gated C1-FFLs are still associated with a successful response to selection for filtering out a short spurious signal. The signal can directly regulate the effector genes. **a** We arbitrarily divide the 36 replicate simulations into high-fitness (red) and low-fitness (blue) groups. **b** The high-fitness replicates still evolve AND-gated C1-FFLs. Bars are mean ± s.e.m. of the occurrence over replicate evolutionary simulations.

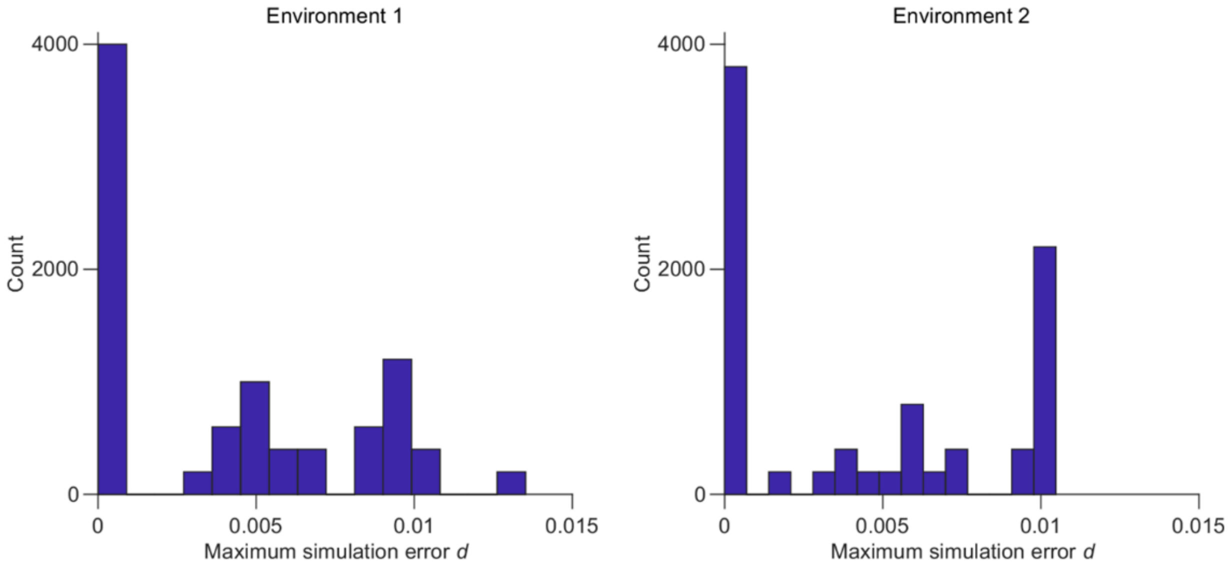

Supplementary Figure 12. Our updating algorithm is able to limit simulation errors. The distribution across 9,000 simulations of the maximum value of simulation error *d* (defined in Supplementary Equation 14) over the course of gene expression. For each of the 45 evolutionary replicates in Fig. 4, we run 200 simulations of gene expression of the final evolved genotype. These genotypes were the outcome of evolution under selection for filtering out short spurious signals, in which direct regulation of the effector by the signal is not allowed. In environment 1 a genotype responds to a constant ON signal and in environment 2 it responds to a short spurious signal (Fig. 3).

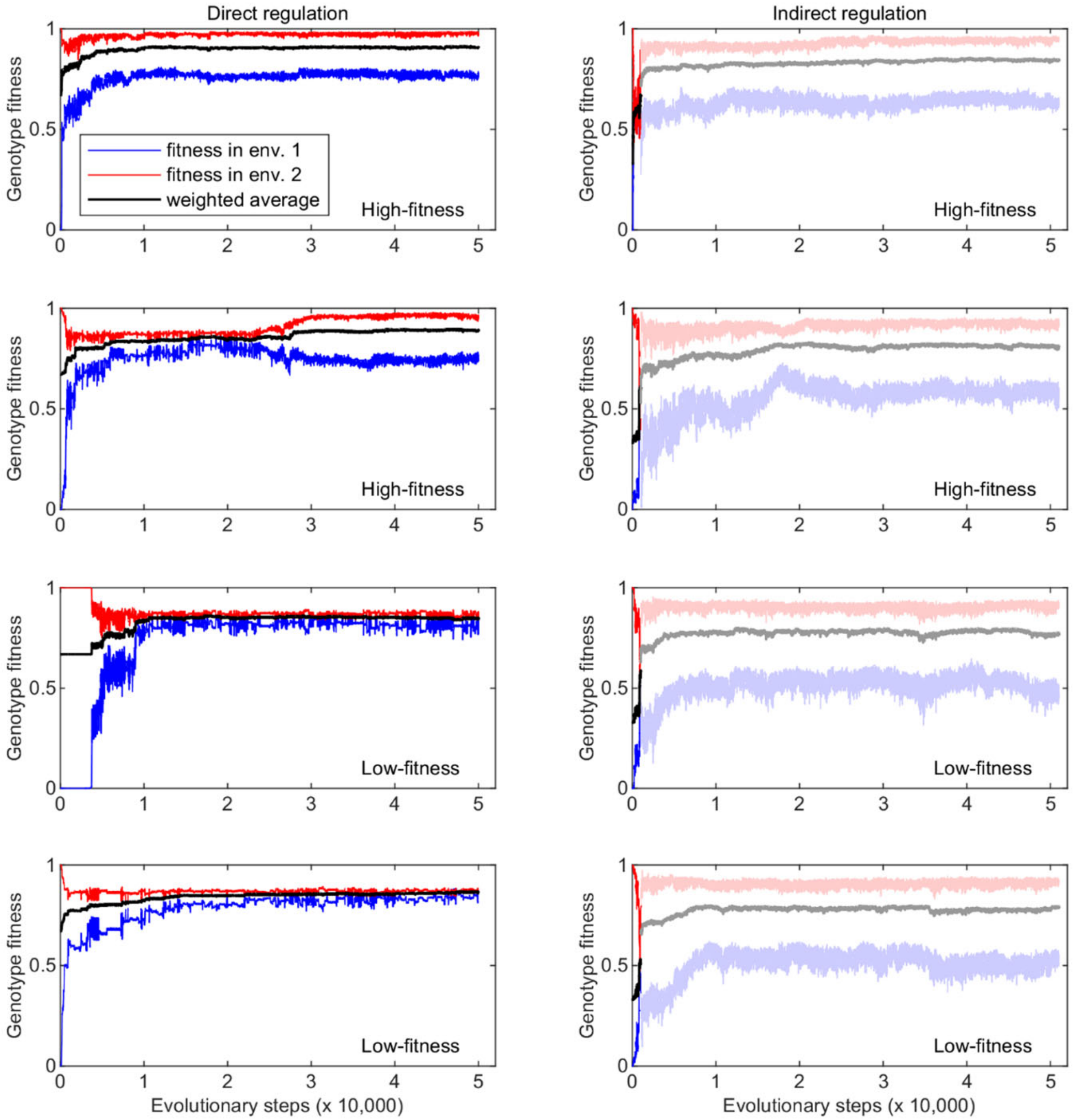

Supplementary Figure 13. Representative fitness trajectories under selection to filter out short spurious signals. Left panels: The signal is allowed to directly regulate the effector genes. Panels 1 and 3 correspond to the two genotypes shown in Supplementary Figure 3. Right panels: the signal cannot directly regulate the effector genes. Average fitness (black) is a weighted average of the blue and red trajectories, with environment 2 (where the signal is spurious) being considered twice as common as environment 1 (where the signal is sustained and real). When the signal cannot directly regulate the effector genes, evolutionary simulations begins with a burn-in phase that lasts 1,000 evolutionary steps (see Evolutionary Simulation in the Methods). We show the burn-in phase in undiluted color, and dilute color after burn-in. Most replicates quickly reach a stable fitness plateau (first and third rows). Certain replicates can be temporarily trapped at a low fitness plateau (second and third rows on the left).

Supplementary Note 1

Hooshangi et al. [23] found that a simple transcriptional cascade (signal -> TF -> effector) could filter out short spurious signals when the intermediate TF is rapidly degraded, dampening the effect of a brief signal. Two such transcriptional cascades involving different intermediate TFs form a diamond, so the utility of a single cascade is a potential explanation for the high prevalence of double-cascade diamonds. However, in this case we would have no reason to expect marked differences in expression dynamics between the two TFs, as illustrated in Fig. 8 and Supplementary Table 4. Enrichment for AND-gates (Fig. 7b, c) indicates selection to integrate information from the two cascades. On the other hand, we do find some non-AND-gated diamonds (Fig. 7b), and these might best be considered as cascades. Inspection of their parameter values reveals that in these diamonds, both TFs have fast-degrading mRNAs and proteins so that both TFs shut down rapidly once signal is turned off. This makes such diamonds less vulnerable to spurious signals, reducing the need for the AND gate. The difficulty of evolving not just one but two fast-degrading high-affinity TFs likely explains why non-AND-gated diamonds are rare. When weak TFBSs are excluded, these non-AND-gated diamonds are nevertheless scored as AND-gated (Fig. 7b).

Supplementary Note 2

To investigate the phenomenon of near-AND-gated motifs, we added a weak (two-mismatch) TFBS to the effector's cis-regulatory region in evolved AND-gated isolated C1-FFLs and diamonds. This converts them to near-AND-gated. This lowers fitness only when the extra link is from the slow TF to the effector (see definition of fast and low TFs in the section "Diamonds also evolve under no external spurious signals" under Results), and not when the extra link is from the fast TF to the effector (Supplementary Figure 7).

Indeed, these extra links are tolerated during evolution too. In Fig. 7c, if we take the 16 high-fitness replicates that contain a near-AND-gated C1-FFL in at least 1% of the evolutionary steps, then for 15 replicates of the 16, at least 88% of the near-AND-gated C1-FFLs in each of the 15 replicates are only near-AND-gated because of extra weak TFBSs for the fast TF. In the remaining 1 replicate, 93% of the near-AND-gated C1-FFLs have extra weak TFBSs specific for each of the TFs (and are therefore scored as OR-gated). In this last replicate, the two TFs in these OR-gated C1-FFLs have high and similar protein degradation rates, reducing the need for an AND gate for reasons discussed earlier. We similarly examine high-fitness replicates that, when upstream weak TFBSs are excluded, contain a near-AND-gated diamond in at least 1% of the evolutionary steps. In 15 of these 24 evolutionary replicates, the near-AND regulatory logic is in most evolutionary steps due to an extra weak TFBS of the fast TF, in 8 replicates (all of them OR-gated, like the OR-gated C1-FFL already discussed) it is due to weak TFBSs for each of the TFs, and in only 1 replicate is it due to an extra TFBS for the slow TF. For the latter two categories, both TFs in near-AND-gated diamonds have high and similar protein degradation rates. By chance alone, fast and slow TF should be equally likely to contribute the weak TFBS that makes a motif near-AND-gated rather than AND-gated. This expected 50:50 ratio can be rejected from our observed 15:0 and 15:1 ratios with $p = 3 \times 10^{-5}$ and $p = 3 \times 10^{-4}$, respectively (cumulative binomial distribution, one-sided test). This non-random occurrence of weak TFBSs creating near-AND-gates illustrates how even weak TFBSs can be shaped by selection against some (but not all) motif-breaking links.

Supplementary Methods

TF binding

Transcription of each gene is controlled by TFBSs present within a 150-bp cis-regulatory region, corresponding to a typical yeast nucleosome-free region within a promoter [1]. The perfect TFBS for a typical yeast TF has information content equivalent to 13.8 bits [2]; this means that in a simplified model of binding where only one of the four nucleotides is a good match at each site, ~7 bp are recognized as an optimal consensus binding site. Maerkl & Quake [6] reported that the TFBSs of two yeast TFs, Pho4p and Cbf1p, can have up to 2 mismatched sites within their 6 bp consensus binding sequence, while still binding the TF above background levels [6]. Our model therefore tracks TFBSs with up to 2 mismatches. This low information content implies a higher density of TFBSs within our cis-regulatory regions than our algorithm was able to handle, so we instead assigned each TF an 8-bp consensus sequence. Two TFs cannot simultaneously occupy overlapping stretches (Supplementary Figure 2), which we assume extend beyond the recognition sequence to occupy a total of 14 bp [3]; this captures competitive binding. The consequences of hindrance between TFBSs for the regulation of effector gene expression are shown in Fig. 2.

We denote the dissociation constant of a TFBS with m mismatches as $K_d(m)$. Sites with $m > 3$ mismatches are assumed to still bind at a background rate equal to $m = 3$ mismatches, with dissociation constant $K_d(3) = 10^{-5}$ mole per liter [6] for all TFs. We assume that each of the last three base pairs makes an equal and independent additive contribution $\Delta G_{bp} < 0$ to the binding energy [24]: although not always true, this approximates average behavior well [6]. We ignore cooperativity in binding. Dissociation constants of eukaryotic TFs for perfect TFBSs can range from 10^{-5} mole per liter [5] to 10^{-11} mole per liter [4]. We initialize each TF with its own value of $\log_{10}(K_d(0))$ sampled from a uniform distribution between -6 and -9, with mutation capable of further expanding this range, subject to $K_d(0) < 10^{-5}$ mole per liter. Substituting $m = 0$ and $m = 3$ into

$$\Delta G_m = -RT\ln K_\mathrm{d}(m) = \Delta G_0 - \min(m,3)\Delta G_\mathrm{bp}, \tag{1}$$

where R is the gas constant and T is temperature, we can solve for ΔG_{bp} and ΔG_0, and thus obtain $K_d(1)$ and $K_d(2)$ (the dissociation constants for TFBS with one and two mismatches, respectively).

Because TFs bind non-specifically to DNA at a high background rate, each nucleosome-free stretch of 14 bp can be considered to be a non-specific binding site (NSBS). A haploid *S. cerevisiae* genome is 12 Mb, 80% of which is wrapped in nucleosomes [25], yielding approximately 10^6 potential non-specific binding sites (NSBSs). In a yeast nucleus of volume 3×10^{-15} liters, the NSBS concentration is of order 10^{-4} mole per liter. To find the concentration of free TF [TF] in the nucleus given a total nucleic TF concentration of C_{TF}, we consider

$$K_\mathrm{d} = \frac{[\mathrm{binding\_site}][\mathrm{TF}]}{[\mathrm{binding\_site{\cdot}TF}]}, \tag{2}$$

in the context of NSBSs, substitute [TF·NSBS] with C_{TF} - [TF], and solve for

$$[\mathrm{TF}] = \frac{K_\mathrm{d}(3)}{K_\mathrm{d}(3)+[\mathrm{NSBS}]} C_\mathrm{TF} = \frac{10^{-5}}{10^{-5}+10^{-4}} C_\mathrm{TF} \approx 0.1 C_\mathrm{TF}. \quad (3)$$

Thus, about 90% of total TFs are bound non-specifically, leaving about 10% free. The relatively small number of specific TFBSs is not enough to significantly perturb the proportion of free TFs, and so for the specific TFBSs with $m < 3$ that are of interest in our model, we simply use $\widehat{K}_\mathrm{d}(m) = 10K_\mathrm{d}(m)$ to account for the reduction in the amount of available TF due to non-specific binding. We also convert $\widehat{K}_\mathrm{d}$ from the units of mole per liter in which K_d is estimated empirically to the more convenient molecules per nucleus. The rescaling factor r for which $\widehat{K}_\mathrm{d}$ (in molecule per nucleus) = $r\widehat{K}_\mathrm{d}$ (in mole per liter) is 3×10^{-15} liter per nucleus $\times\, 6.02 \times 10^{23}$ molecule mole^{-1} = 1.8×10^{9} molecule cell^{-1} liter mole^{-1}. Taken together, $\widehat{K}_\mathrm{d}$ (molecule per nucleus) = $10rK_\mathrm{d}$ (mole per liter), where the factor 10 accounts for non-specific TF binding.

TF occupancy

Here we calculate the probability that there are A activators and R repressors bound to a given cis-regulatory region at a given moment in gene expression time. First we note that if we consider TF i binding to TFBS j in isolation from all other TFs and TFBSs, Supplementary Equation 4 gives us probability of being bound:

$$P_\mathrm{b}(j) = 1 - P_\mathrm{u}(j) = \frac{C_i}{\widehat{K}_\mathrm{d}+C_i} \quad (4)$$

Let $P_{A,R}^{(n)}$ be a term proportional (for a given value of n) to the combined probability of all binding configurations in which exactly A activators and R repressors are bound to the first n binding sites along the cis-regulatory sequence. We calculate $P_{A,R}^{(n)}$ recursively, considering one additional TFBS at each step. Note that if two different TFs bind to exactly the same location on a cis-regulatory region, we treat this as two TFBSs, not as one, and treat first one and then the other in our recursive algorithm.

Consider the case where the (n+1)th binding site belongs to an activator. The case where this activator is not bound contributes $P_{A,R}^{(n)} P_\mathrm{u}(n+1)$ to $P_{A,R}^{(n+1)}$. If it is bound, then we must also take into account that the (n+1)th binding site overlaps (partially or completely) with the last $H \geq 0$ sites, and so contribute $P_{A-1,R}^{(n-H)} P_\mathrm{b}(n+1) \prod_{j=n-H+1}^{n} P_\mathrm{u}(j)$. Taken together,

$$P_{A,R}^{(n+1)} = P_{A,R}^{(n)} P_\mathrm{u}(n+1) + P_{A-1,R}^{(n-H)} P_\mathrm{b}(n+1) \prod_{j=n-H+1}^{n} P_\mathrm{u}(j). \quad (5)$$

Similarly, if the (n+1)th site belongs to a repressor, we have

$$P_{A,R}^{(n+1)} = P_{A,R}^{(n)} P_\mathrm{u}(n+1) + P_{A,R-1}^{(n-H)} P_\mathrm{b}(n+1) \prod_{j=n-H+1}^{n} P_\mathrm{u}(j). \quad (6)$$

By definition, $P_{A,R}^{(n)} = 0$ for binding configurations that are impossible, e.g. those with negative A or negative R. We initialize the recursion at n = 0, where the only valid binding configuration is for A = R = 0, i.e. $P_{0,0}^{(0)} = 1$. At n = 1, $P_{0,0}^{(1)} \propto P_\mathrm{u}(1)$ and if the binding site belongs to an activator

$P_{1,0}^{(1)} \propto P_{\mathrm{b}}(1)$; otherwise, $P_{1,0}^{(1)} \propto P_{\mathrm{b}}(1)$. For a gene where the total number N of TFBSs is 1, $P_{0,0}^{(1)}$, $P_{1,0}^{(1)}$, and $P_{0,1}^{(1)}$ sum to 1 and normalization is unnecessary. For higher values of $N = N_{\mathrm{Act}} + N_{\mathrm{Rep}}$ TFBSs, where N_{Act} and N_{Rep} are the total numbers of activator binding sites and repressor binding sites, respectively, we normalize $P_{A,R}^{(N)}$ at the end of the recursion by dividing by $\sum_{A=0}^{N_{\mathrm{Act}}} \sum_{R=0}^{N_{\mathrm{Rep}}} P_{A,R}^{(N)}$ to get the probability of binding configurations that include exactly A activators and R repressors.

$r_{\mathrm{Act\_to\_Int}}$

Transcription initiation over an interval of time $r_{\mathrm{transc\_init}}$ is proportional to the proportion of time spent in the Active state. Assuming a steady state between Repressed, Intermediate, and Active states, as a function of current TF concentrations, we have:

$$\frac{r_{\mathrm{transc\_init}}}{r_{\mathrm{max\_transc\_init}}} = \frac{r_{\mathrm{Int\_to\_Act}}}{r_{\mathrm{Int\_to\_Act}} + r_{\mathrm{Act\_to\_Int}}} P_{\mathrm{Int\_or\_Act}}, \tag{7}$$

where $P_{\mathrm{Int\_or\_Act}}$ is the probability a gene is at Intermediate or Active. We set $r_{\mathrm{max\_transc\_init}}$ (the rate of transcription given 100% Active state) to 6.75 min^{-1}, based on the corresponding rate when a model of the *PHO5* promoter is fit to data [8]. In this model fit, the constitutively expressed *PHO5* promoter is free of nucleosomes 80% of the time, i.e. $P_{\mathrm{Int\_or\_Act}} = 0.8$. We take these two values as universal for constitutively expressed genes, and assume that variation in $r_{\mathrm{Act\_to\_Int}}$ is responsible for variation in $r_{\mathrm{transc\_init}}$. To identify a set of constitutively expressed genes, we identified 225 genes that have mRNA production rate of at least 0.5 molecule min^{-1} from genome-wide measurements [10]; this threshold corresponds to low H2A.Z occupancy [9]. We set $r_{\mathrm{transc\_init}}$ to the production rate of mRNA of these 225 genes, and solve for gene-specific $r_{\mathrm{Act\_to\_Int}}$ from Eq. S2. We fit the solutions to a log-normal distribution and arrive at $10^{N(1.27,\, 0.226)}$ min^{-1}.

To initialize values of $r_{\mathrm{Act\_to\_Int}}$ for each gene, we sample from this distribution. We also set lower and upper bounds for allowable values; if either the initial sample or subsequent mutation put $r_{\mathrm{Act\_to\_Int}}$ beyond these bounds, we set the value of $r_{\mathrm{Act\_to\_Int}}$ to equal to boundary value. We set the lower bound for $r_{\mathrm{Act\_to\_Int}}$ at 0.59 min^{-1}, half the minimum of the values inferred from the set of 225 genes. To set an upper bound, we use the low H2A.Z occupancy bound of $r_{\mathrm{transc\_init}} = 0.5$, which gives a solution of 32.34 min^{-1}; we double this to set the upper bound as 64.7 min^{-1}.

Transcription delay times

Yeast protein lengths fit a log-normal distribution of $10^{N(2.568,\, 0.34)}$ amino acids (from the Saccharomyces Genome Database [11], excluding mitochondrial proteins; YeastMine [12] was used to query the database and to download data). We sample ORF length L from this distribution. To constrain the values of L, we set a lower bound of 50 amino acids and an upper bound of 5,000 amino acids; the longest protein in SGD is 4910 amino acids. If either initialization or mutation put L beyond these bounds, we set the value of L to the boundary value.

With an mRNA elongation rate of 600 codon per min [14, 15], it takes L / 600 minutes to transcribe the ORF of an mRNA. Also including time for transcribing UTRs and for transcription termination, and ignoring introns for simplicity, it takes 290 seconds to complete transcription of the yeast *GLT1* gene [15], whose ORF is 6.4kb. Putting the two together, we infer that

transcribing the UTRs and terminating transcription takes around 1 minute for *GLT1*. Generalizing to assume that transcribing UTRs and terminating transcription takes exactly 1 minute for all genes, producing an mRNA from a gene of length L takes $1 + L / 600$ minutes.

Translation delay times and $r_{protein\_syn}$

We model a second delay between the completion of a transcript and the production of the first protein from it. The delay comes from a combination of translation initiation and elongation; it ends when the mRNA is fully loaded with ribosomes all the way through to the stop codon and the first protein is produced. We ignore the time required for mRNA splicing; introns are rare in yeast [13]. mRNA transportation from nucleus to cytosol, which is likely diffusion-limited [26, 27], is fast even in mammalian cells [28] let alone much smaller yeast cells, and the time it takes is also ignored. The median time in yeast for initiating translation is 0.5 minute [Table 1 in 17], and the genomic average peptide elongation rate is 330 codon/min [17]. After an mRNA is produced, we therefore wait for $0.5 + L / 330$ minutes, and then model protein production as continuous at a gene-specific rate $r_{protein\_syn}$.

To calculate $r_{protein\_syn}$, we combine the gene-specific ribosome densities D along the mRNAs and the gene-specific peptide elongation rates E, both measured in yeast [17]. The values of DE across yeast genes fit the log-normal distribution $10^{N(0.322,\, 0.416)}$ molecule $mRNA^{-1}$ min^{-1}; we initialize $r_{protein\_syn}$ for each gene by sampling from this distribution. We set the lower bound for $r_{protein\_syn}$ at half the minimum observed value of DE (4.5×10^{-3} molecule $mRNA^{-1}$ min^{-1}). The upper bound corresponds to an mRNA fully occupied by rapidly moving ribosomes. Each ribosome occupies about 10 codons [17], and the peptide elongation rate can be as high as 614 codon per min [29]. If ribosomes are packed closely together at 10 codons apart, a protein comes off the end of production in the time taken to elongate 10 codons, i.e. proteins are produced at 61.4 molecules per minute. If either initialization or mutation put $r_{protein\_syn}$ beyond these bounds, we set the value of $r_{protein\_syn}$ to the boundary value.

mRNA and protein decay rates

We fit the log-normal distribution $10^{N(-1.49,\, 0.267)}$ min^{-1} to yeast mRNA degradation rates [16], and initialize the mRNA degradation rate r_{mRNA_deg} for each gene by sampling from this distribution. We set lower and upper bounds for r_{mRNA_deg} at half the minimum and twice the maximum observed values (7.5×10^{-4} min^{-1} and 0.54 min^{-1}), respectively. If either initialization or mutation put r_{mRNA_deg} beyond these bounds, we set the value of r_{mRNA_deg} to the boundary value.

Expressing the estimated half-lives of yeast proteins [18] in terms of protein degradation rates, they fit the log-normal distribution $10^{N(-1.88,\, 0.56)}$ min^{-1}; we initialize gene-specific protein degradation rates $r_{protein\_deg}$ by sampling from this distribution. We ignore the additional reduction in protein concentration due to dilution as the cell grows and thus increases in volume. We set lower and upper bounds for $r_{protein\_deg}$ at half the minimum and twice the maximum observed degradation rate (3×10^{-6} min^{-1} and 0.69 min^{-1}), respectively. If either initialization or mutation put $r_{protein\_deg}$ beyond these bounds, we set the value of $r_{protein\_deg}$ to the boundary value.

Simulation of gene expression

Our algorithm is part-stochastic, part-deterministic. We use a Gillespie algorithm [30] to simulate stochastic transitions between Repressed, Intermediate, and Active chromatin states, and to simulate transcription initiation and mRNA decay events. We refer to these as "Gillespie

events". The completion of transcription to produce a complete mRNA, and subsequent ribosomal loading onto the mRNA, are referred to as "fixed events" (they require fixed times of 1 + *L* / 600 minutes and 0.5 + *L* / 330 minutes, respectively). Scheduled changes in the strength of the external signal are also fixed events. Protein production and degradation are described deterministically with ODEs, and updated frequently in order to recalculate TF concentrations and hence chromatic transition rates. Updates occur at the time of Gillespie and fixed events, and also in between.

The total rate of all Gillespie events is

$$r_{\text{total}} = \sum_{i=1}^{\text{Rep}} r_{\text{Rep\_to\_Int\_i}} + \sum_{i=1}^{\text{Int}} (r_{\text{Int\_to\_Rep\_i}} + r_{\text{Int\_to\_Act\_i}}) + \sum_{i=1}^{\text{Act}} (r_{\text{Act\_to\_Int\_i}} + r_{\text{transc}}) + \sum_{i=1}^{N_{\text{copies}}} r_{\text{mRNA\_deg\_i}} N_{\text{mRNA\_i}}, \quad (8)$$

where Rep, Int, and Act are the numbers of gene copies in our haploid model that are in the Repressed, Intermediate, and Active chromatin states, respectively, $N_{\text{mRNA\_i}}$ is the number of completely transcribed mRNA molecules from gene *i*, and N_{copies} is the total number of gene copies. We only simulate degradation of full transcribed mRNA, and not that of mRNA that are still being transcribed, because the latter are already captured implicitly by $r_{\text{max\_transc\_init}}$, which is based on mRNAs that complete transcription [8]. Once an mRNA finishes transcription, it is subjected to degradation regardless of whether ribosome loading is complete.

The waiting time Δt_G before the next Gillespie event is

$$\Delta t_{\text{G}} = \frac{x}{r_{\text{total}}}, \quad (9)$$

where *x* is random number drawn from an exponential distribution with mean 1. Which Gillespie event takes place next is sampled only if a different update does not happen first. If a fixed event is scheduled to happen first at $\Delta t_F < \Delta t_G$, we advance time by Δt_F, update the state of the cell, and calculate a new r_{total}'. Since the cellular activity has been going on with the old rate r_{total} for Δt_F, the remaining "labor" required to trigger the Gillespie event planned earlier is reduced. The new waiting time, $\Delta t_G'$, to trigger the planned Gillespie event is

$$\Delta t_{\text{G}}' = \frac{x - r_{\text{total}} \Delta t_{\text{F}}}{r_{\text{total}}'}. \quad (10)$$

Gene duplication creates $n \geq 1$ genes copies producing the same protein, where each copy *i* might have diverged in their production rate $r_{\text{protein\_syn\_i}}$ and degradation rate $r_{\text{protein\_deg\_i}}$. Complete proteins are produced continuously once an mRNA molecule is fully loaded with ribosomes, which occurs 0.5 + *L* / 330 minutes after transcription is complete – the concentration of such molecules is denoted $N_{\text{mRNA\_aft\_delay\_i}}(t)$. The total concentration of a protein obeys:

$$N'_{\text{protein}}(t) = \sum_{i}^{n} \left(r_{\text{protein\_syn\_i}} N_{\text{mRNA\_aft\_delay\_i}}(t) - r_{\text{protein\_deg\_i}} N_{\text{protein\_i}}(t) \right) \quad (11)$$

Protein concentrations are updated using a closed-form integral of Supplementary Equation 11

$$N_{\text{protein}}(t_1) = \sum_i^n \left(\frac{r_{\text{protein\_syn\_i}} N_{\text{mRNA\_aft\_delay\_i}}}{r_{\text{protein\_deg\_i}}} + \left(N_{\text{protein\_i}}(t_0) - \frac{r_{\text{protein\_syn\_i}} N_{\text{mRNA\_aft\_delay\_i}}}{r_{\text{protein\_deg\_i}}}\right) \mathrm{e}^{-r_{\text{protein\_deg\_i}}(t_1 - t_0)}\right) \quad (12)$$

with this expression updated every time a Gillespie or fixed event at time t_1 changes the value of $N_{\text{mRNA\_aft\_delay\_i}}$.

In between updates, values of P_{A}, P_{R}, $P_{\text{A\_no\_R}}$, and $P_{\text{notA\_no\_R}}$, and hence chromatin transition rates, are calculated under the approximation of constant N_{protein}. Additional updates, above and beyond fixed and Gillespie events, are performed in order to ensure that chromatin transition rates do not change too dramatically from one update to the next. We use a target of $D = 0.01$ for the amount of change tolerated in the values of P_{A}, P_{R}, $P_{\text{A\_no\_R}}$, and $P_{\text{notA\_no\_R}}$, in order to schedule updates after time Δt_{U}, which are triggered when neither a Gillespie event nor a fixed event occurs before this time has elapsed, i.e. when $\Delta t_{\text{U}} < \Delta t_{\text{F}}$ and $\Delta t_{\text{U}} < \Delta t_{\text{G}}$.

There is the greatest potential for large changes after an update that changes the value of $N_{\text{mRNA\_aft\_delay\_i}}$. In this case, we use Supplementary Equation 4 to solve for the time interval for which the probability that TF *i* would be bound to a single perfect and non-overlapping TFBS would change by D, by choosing $\Delta t_{\text{U}} > 0$ that satisfies

$$\left|\frac{N_i(t)}{N_i(t)+\widehat{K}_{\text{d\_i}}(0)} - \frac{N_i(t+\Delta t_{\text{U}})}{N_i(t+\Delta t_{\text{U}})+\widehat{K}_{\text{d\_i}}(0)}\right| = D. \quad (13)$$

A solution for Δt_{U} may not exist, e.g. if the concentration of TF *i* is decreasing but $P_{\text{b\_i}}(t) < D$. In such cases, we set Δt_{U} to infinity.

When the previous update does not change any $N_{\text{mRNA\_aft\_delay\_i}}$ values, then we modify Δt_{U} adaptively. Let d be the maximum of ΔP_{A}, ΔP_{R}, $\Delta P_{\text{A\_no\_R}}$, and $\Delta P_{\text{notA\_no\_R}}$ during the last update, and Δt be the advance in time between the last two updates. We then schedule an update at

$$\Delta t_{\text{U}}' = \frac{D}{d} \Delta t. \quad (14)$$

After an update that changes the value of $N_{\text{mRNA\_aft\_delay\_i}}$, we use the smaller value from Supplementary Equations 13 and 14. These additional update times are discarded and recalculated when a Gillespie or fixed event occurs first.

In Supplementary Figure 12, we see that simulations rarely exceed our target of $D = 0.01$, and do so only modestly.

Cost of gene expression

The cost of gene expression comes from some combination of the act of expression and from the presence of the resulting gene product. Yeast cells with plasmids carrying fast-degrading GFP had as much growth impairment as those carrying wild-type GFP [Fig. 3 of 20], suggesting that the former cost dominates. Universal costs stemming from the act of gene expression include the consumption of energy [31, 32] and the opportunity cost of not using ribosomes to make other gene products [33]. While some costs arise from transcription [20], we simplify our model by attributing all of the cost of expression to the act of translation.

Kafri et al. [20] reported that, in rich media, the growth rate of haploid yeast is reduced by about 1% when mCherry is expressed to about 2% of proteome. With $b_{max} = 1$ giving the growth rate of the yeast when mCherry is not expressed, we have the cost of gene expression equal to 0.01. Next, we estimate the production rate of mCherry in Kafri et al. [20] by assuming that mCherry is in steady state between production and dilution due to cell division; fluorescent proteins tend to be stable such that degradation can be ignored [34]. Ghaemmaghami et al. [19] estimated that a haploid yeast cell contains about 5×10^7 protein molecules, 2% of which are now mCherry. Over a 90 minute cell cycle in Kafri et al. [20], about 5×10^5 mCherry molecule per cell need to be expressed in order to double in numbers. This yields a production rate of about 5×10^3 mCherry molecules per minute per cell. Because the total cost of gene expression is 0.01, the cost at a protein production rate of one mCherry molecule per minute per cell, c_{transl}, is 2×10^6. Long genes should be more expensive to express than short ones; for a gene of length L, we assume its cost of expression is $c_{transl}L$ / 370, where 370 is the geometric mean length of a yeast protein as described above in "Transcription delay times". Results using the length of mCherry instead, i.e. a slightly higher cost of expression of $c_{transl}L$ / 236, are unlikely to be significantly different.

The overall cost of gene expression at time t, $C(t)$ is:

$$C(t) = c_{\text{transl}}\left(\sum_{i=1}^{N_{\text{copies}}} \frac{L_i}{10^{2.568}} r_{\text{transl\_init\_i}} N_{\text{mRNA\_aft\_delay\_i}}(t) + \sum_{i=1}^{N_{\text{copies}}} \frac{L_i}{10^{2.568}} \frac{r_{\text{transl\_init\_i}}}{2} N_{\text{mRNA\_during\_delay\_i}}(t)\right). \quad (15)$$

The second term represents transcripts that are on average half-loaded with ribosomes, and hence experiencing on average half the cost of translation. We integrate $C(t)$ within segments of constant $C(t)$ to obtain the overall cost of gene expression during a simulation.

Mutation

Because we use an origin-fixation approach, only the relative and not the absolute values of our mutation rates matter. In *S. cerevisiae,* the rates of small indels and of single nucleotide substitutions have been estimated as 0.2×10^{-10} per base pair and 3.3×10^{-10} per base pair, respectively [35]. Thus, cis-regulatory sequences are primarily shaped by single nucleotide substitutions. We do not model small indels in the cis-regulatory sequence, but increase the single nucleotide substitution up to 3.5×10^{-10} per base pair to compensate. This corresponds to a rate of 5.25×10^{-8} per 150 bp cis-regulatory sequence.

Lynch et al. [35] also report a rate of gene duplication of 1.5×10^{-6} per gene and of deletion of 1.3×10^{-6} per gene (not including non-deletion-based loss of function mutations). These values turned out to swamp the evolution of TFBSs and hence significantly slow down our simulations, so we chose values 10-fold lower, making both gene duplication and gene deletion occur at rate 1.5×10^{-7} per gene. This preserves their numerical excess but reduces its magnitude.

Our model contains 8 gene-specific parameters, namely L, $r_{\text{Act\_to\_Int}}$, $r_{\text{protein\_deg}}$, $r_{\text{protein\_syn}}$, $r_{\text{mRNA\_deg}}$, the $K_d(0)$ of a TF, whether a TF is an activator vs. repressor, and the consensus binding sequence of a TF. We assume mutations to L are caused by relatively neutral small indels, which we assume to be 20% of all small indels; mutation to L therefore occurs at rate 1.2×10^{-11} per codon, i.e. $1.2 \times 10^{-11}L$ for a gene of length L. For $r_{\text{Act\_to\_Int}}$, we assume that it is altered by 10%

of all the point mutations (single nucleotide substitution and small indels) to the core promoter of a gene. The length of a core promoter is about 100 bp and is relatively constant among genes [36], yielding a mutation rate of $r_{\mathrm{Act\_to\_Int}}$ of 3.5×10^{-9} per gene.

The remaining 6 gene-specific parameter mutation rates are parameterized with lower accuracy due to lack to data; the principal decision is which to make dependent vs. independent of gene length. TF binding to DNA depends on particular peptide motifs whose length is likely independent of TF length, therefore we make mutation rates independent of gene length for mutations to $K_{\mathrm{d}}(0)$, to the consensus binding sequence of a TF, and to the activating vs repressing identity of a TF. We set the rate of each of the three mutation types to 3.5×10^{-9} per gene. In contrast, because the stability of an mRNA mainly depends on its codon usage [37] and thus more codons means more opportunities for change, we assume the rate of mutation to $r_{\mathrm{mRNA\_deg}}$ does depend on gene length, as do mutations to protein stability $r_{\mathrm{protein\_deg}}$. $r_{\mathrm{protein\_syn}}$ is determined by the density of ribosomes on mRNA and the elongation rate of ribosomes, and therefore is affected both by ribosome loading speed and by slow spots forming queues in the mRNA. Ribosome loading often relies on the 5'UTR of mRNA [38], and 5'UTR length is positively correlated with ORF length [39]. Slow-spots in mRNA can be due to secondary structure or to suboptimal codons, therefore are also more likely to appear by mutation to long mRNAs, so we assume the rate of mutation to $r_{\mathrm{protein\_syn}}$ depends on gene length. We set the mutation rates of $r_{\mathrm{protein\_deg}}$, $r_{\mathrm{protein\_syn}}$, and $r_{\mathrm{mRNA\_deg}}$ each to 9.5×10^{-12} per codon; in other words, each mutation rate is 3.5×10^{-9} for a yeast gene of average length (on a log-scale) $10^{2.568}$ = 370 codons.

$r_{\mathrm{Act\_to\_Int}}$, $r_{\mathrm{protein\_syn}}$, $K_{\mathrm{d}}(0)$, $r_{\mathrm{protein\_deg}}$, and $r_{\mathrm{mRNA\_deg}}$ evolve as quantitative traits. They are assumed to have, in the absence of selection, a log-normal stationary distribution with mean μ and standard deviation σ, with values estimated below and listed in Supplementary Table 2. Denote the values of a parameter as *x* before mutation and *x'* after mutation; mutation takes the form:

$$\log_{10}x' = \log_{10}x + \mathrm{Normal}(k(\mu - \log_{10}x), \sigma), \quad (16)$$

where *k* controls the speed of regressing back to the stationary distribution; we set *k* = 0.5 for all 5 parameters. To set values of μ, central tendency estimates of these five values (from Supplementary Table 1**)** are adjusted according to our expectations about mutation bias. We assume a mutation bias toward faster mRNA degradation $r_{\mathrm{mRNA\_deg}}$, faster $r_{\mathrm{Act\_to\_Int}}$ [36, 40], slower translation initiation $r_{\mathrm{protein\_syn}}$ [38], and larger $K_{\mathrm{d}}(0)$. We assume that the observed log-normal means of $r_{\mathrm{mRNA\_deg}}$, $r_{\mathrm{protein\_syn}}$, and $r_{\mathrm{Act\_to\_Int}}$ differ by 2-fold from the mean expected from mutational bias; for example, the mean of $\log_{10}(r_{\mathrm{mRNA\_deg}})$ is -1.49, so the value of μ for $r_{\mathrm{mRNA\_deg}}$ is -1.49 + $\log_{10}(2)$ = -1.19. We assume a larger bias for $K_{\mathrm{d}}(0)$, namely that mutation is likely to reduce the affinity of a TF for a TFBS down to non-specific levels. Thus, we set $\mu = \log_{10}(K_{\mathrm{d}}(3))$ = -5 for $K_{\mathrm{d}}(0)$; note that in this case μ is equal to one of the boundary values, which will be hit far more often than during the evolution of other parameters. We assume that the observed central tendency estimate of protein stability does not depart from mutational equilibrium, therefore the value of μ for $r_{\mathrm{protein\_deg}}$ is the mean of $\log_{10}(r_{\mathrm{protein\_deg}})$ = -1.88.

The value of σ controls mutational effect size. We set the value of σ such that 1% of mutational changes from $x = 10^{\mu}$ go beyond the boundary values, for simplicity approximating by considering only the closer of the two boundary values on a log scale, i.e. we solve Supplementary Equation 17 for σ:

$$\begin{cases} P(\mu + \text{Normal}(0,\sigma) \geq \log_{10} B_\text{U}) = 0.01, \text{if the upper bound } B_\text{U} \text{ is closer} \\ P(\mu + \text{Normal}(0,\sigma) \leq \log_{10} B_\text{L}) = 0.01, \text{if the lower bound } B_\text{L} \text{ is closer} \end{cases} \quad (17)$$

For example, the upper and the lower bounds of $r_{\text{mRNA\_deg}}$ are 0.54 min^{-1} and 7.5×10^{-4} min^{-1}; on a log-scale, the upper bound is closer to $10^{\mu} = 10^{-1.19}$ min^{-1}. Plugging these values in Eq. S8 and solving for σ, we have $\sigma = 0.396$. We set the values of σ for $r_{\text{protein\_syn}}$, and $r_{\text{protein\_deg}}$ in the same way. However for $r_{\text{Act\_to\_Int}}$, σ is set according to the lower bound, even though it is the more distant from 10^{μ}, because otherwise a stable preinitiation complex will evolve too rarely. Under this high mutational variance, evolutionary outcomes at the two bounds are still only observed 5% of the time. For $K_\text{d}(0)$, because its upper bound is equal to 10^{μ}, we set σ to 0.776, such that 1% of mutations can change the values of $K_\text{d}(0)$ by 100-fold or more.

Mutant values of L, $r_{\text{Act\_to\_Int}}$, $r_{\text{protein\_syn}}$, $r_{\text{protein\_deg}}$, and $r_{\text{mRNA\_deg}}$ are constrained by the same bounds that constrain the initial values of these parameters (see previous sections). If a mutation increases the value of any of these 5 parameters to beyond the corresponding upper bound, we set the mutant value to the upper bound; similarly for a mutant value that is smaller than the lower bound of the corresponding parameter. For mutation to $K_\text{d}(0)$, we resample if $x' \geq K_\text{d}(3)$, because otherwise the mutation effectively "deletes" the TF by reducing its affinity to non-specific levels.

Burn-in evolutionary simulation conditions

When the signal is not allowed to regulate the effector genes directly, most simulations under selection either to filter out short spurious signals or for simple signal recognition in the absence of spurious signals rapidly found a local optimal solution in which effector genes are never expressed. This local optimum exists in part because we assume that the environment in which the effector is deleterious is twice as common as the environment in which it is beneficial (Fig. 3). When the signal is not allowed to directly turn on the effector, then to escape this local optimum, at least one activator must be induced by the signal and then induce the effector. Such activators are rare when genotypes are randomly initialized. Making matters worse, mutation tends to reduce expression after initialization (see "Mutation").

To reduce the frequency of this problem, we added a burn-in stage to simulations in which the signal is not allowed to regulate the effector directly. During burn-in, we switch the frequencies of the two environments, so that selection to express the effector is stronger. We also change the mutational bias in $r_{\text{Act\_to\_Int}}$, $r_{\text{protein\_syn}}$, and $K_\text{d}(0)$ to favor higher expression and stronger binding. For $r_{\text{Act\_to\_Int}}$ and $r_{\text{protein\_syn}}$, we use 0.1 instead of 0.01 as the tolerated fraction of extreme mutations in Supplementary Equation 17. For $K_\text{d}(0)$, we decrease μ from -5 to -7.5, biasing mutation toward the mean value at which we initialize (Supplementary Table 1). Evolving an activator that can reliably turn on the effector when the signal is on primarily relies on forming strong binding sites and appropriate kinetic constants in expression, assisted by the change in mutational bias above. To better focus the simulations on sampling appropriate mutations during the burn-in phase, we reduce the rate of gene duplication and the rate of deletion to 5.25×10^{-9} per gene, and limit the maximum number of TF genes to 9 and that of effector genes to 2. Each simulation is run under burn-in conditions for 1,000 steps, after which normal model settings and selection conditions are restored. The same burn-in mutational settings are used for the control selection conditions (no selection, no spurious signal, and harmless spurious signal).

Quantifying occurrence of network motifs

Scoring the presence of a C1-FFL motif (e.g. Fig. 4b) or diamond motif (e.g. Fig. 7) is based on scoring whether TF A regulates gene B. Gene duplication and divergence complicate this scoring, because different gene copies might encode functionally identical proteins, but one copy of gene B might have a TFBS for TF A and the other might not. For the purpose of scoring motifs, our algorithm begins by simply treating each gene copy as though it were a unique gene.

Following Milo et al. [21], a C1-FFL is scored if activating TF A can bind to the cis-regulatory sequence of activating TF B and to the effector, if B can also bind to that of the effector, and if B does not bind to that of A. Auto-regulation is allowed. We exclude C1-FFLs in which A and B encode the same TF or variants of the same TF. In the case of direct regulation, A can be the signal rather than a TF. C1-FFLs can then be subdivided into categories based on overlap between the TFBSs in the cis-regulatory region of the effector (Fig. 2).

A diamond is scored if two signal-regulated activating TFs, A and B, do not bind to each other's cis-regulatory region, but both bind to that of the effector. We allow auto-regulation and require A and B to not encode the same TF or variants of the same TF.

A FFL-in-diamond is scored if one signal-regulated activating TF A binds to the cis-regulatory region of another signal-regulated activating TF B, but B does not bind to that of A, and both A and B bind to that of the effector. Again, auto-regulation is allowed, and A and B must not encode the same TF or variants of the same TF.

Occurrence within one evolutionary replicate is calculated as the fraction of the last 10,000 evolutionary steps in which at least one motif of the type of interest is present. The mean and standard error of this occurrence metric is then calculated across replicates.

Perturbing network motifs

In Fig. 5 and Fig. 9, we add a TFBS to the cis-regulatory sequence of the effector gene, in order to destroy the AND-gate logic of an isolated C1-FFL or diamond. The new TFBS is chosen such that it does not overlap with any existing TFBSs, and has the same affinity as the strongest TFBS that is already present in the cis-regulatory sequence of the effector gene for the signal/fast TF (to convert from an AND-gate to signal-controlled/fast TF-controlled), or for the slow TF (to convert from an AND-gate to slow TF-controlled).

When a TRN has multiple AND-gated motifs of interest, we convert all of them. A perturbation can also affect the logic of other, potentially non-AND-gated motifs in the same TRN (e.g. Supplementary Figure 4), making it hard to attribute the fitness effect to the AND-gate logic of the targeted motif. For this reason, we perform the perturbation analysis not on a single potentially problematic genotype, but on the last 10,000 evolutionary steps of an evolutionary simulation. Within those 10,000 related genotypes, we exclude those that also contain other motifs that might influence our results. For simulations where the signal is allowed to directly regulate the effector, this means excluding those with non-AND-gated C1-FFLs. For simulations where the signal is not allowed to directly regulate the effector, we exclude genotypes with either AND-gated or non-AND-gated motifs other than those of interest (e.g. if we intend to perturb AND-gated isolated C1-FFLs, we exclude genotypes that also contain either an AND-gated isolated diamond or a non-AND-gated C1-FFL). Both pre-perturbation fitness and post-perturbation fitness are averaged over the remaining genotypes. If no evolutionary step meets

our requirement, we exclude the entire evolutionary simulation; this occurs only when the signal cannot directly regulate the effector genes.

Supplementary References

1. Yuan GC, Liu YJ, Dion MF, Slack MD, Wu LF, Altschuler SJ, et al. Genome-scale identification of nucleosome positions in *S. cerevisiae*. Science. 2005;309:626-630.

2. Wunderlich Z, Mirny LA. Different gene regulation strategies revealed by analysis of binding motifs. Trends Genet. 2009;25:434-440. doi: 10.1016/j.tig.2009.08.003.

3. Zhu J, Zhang MQ. SCPD: a promoter database of the yeast *Saccharomyces cerevisiae*. Bioinformatics. 1999;15:607-611. doi: 10.1093/bioinformatics/15.7.607.

4. Nalefski EA, Nebelitsky E, Lloyd JA, Gullans SR. Single-molecule detection of transcription factor binding to DNA in real time: Specificity, equilibrium, and kinetic parameters. Biochemistry. 2006;45:13794-13806. doi: 10.1021/bi0602011.

5. Park S, Chung S, Kim KM, Jung KC, Park C, Hahm ER, et al. Determination of binding constant of transcription factor myc-max/max-max and E-box DNA: The effect of inhibitors on the binding. Biochim Biophys Acta, Gen Subj. 2004;1670:217-228. doi: 10.1016/j.bbagen.2003.12.007.

6. Maerkl SJ, Quake SR. A Systems Approach to Measuring the Binding Energy Landscapes of Transcription Factors. Science. 2007;315:233-237. doi: 10.1126/science.1131007.

7. Katan-Khaykovich Y, Struhl K. Dynamics of global histone acetylation and deacetylation in vivo: rapid restoration of normal histone acetylation status upon removal of activators and repressors. Genes Dev. 2002;16:743-52. doi: 10.1101/gad.967302.

8. Brown CR, Mao C, Falkovskaia E, Jurica MS, Boeger H. Linking Stochastic Fluctuations in Chromatin Structure and Gene Expression. PLoS Biol. 2013;11:e1001621. doi: 10.1371/journal.pbio.1001621.

9. Guillemette B, Bataille AR, Gevry N, Adam M, Blanchette M, Robert F, et al. Variant histone H2A.Z is globally localized to the promoters of inactive yeast genes and regulates nucleosome positioning. PLoS Biol. 2005;3:e384.

10. Pelechano V, Chávez S, Pérez-Ortín JE. A Complete Set of Nascent Transcription Rates for Yeast Genes. PLoS ONE. 2010;5:e115560.

11. SGD Project. [cited 2018 April 2]. Available from: https://yeastmine.yeastgenome.org.

12. Balakrishnan R, Park J, Karra K, Hitz BC, Binkley G, Hong EL, et al. YeastMine—an integrated data warehouse for *Saccharomyces cerevisiae* data as a multipurpose tool-kit. Database. 2012:bar062. doi: 10.1093/database/bar062.

13. Dujon B. The yeast genome project: what did we learn? Trends Genet. 1996;12:263-270. doi: 10.1016/0168-9525(96)10027-5.

14. Hocine S, Raymond P, Zenklusen D, Chao JA, Singer RH. Single-molecule analysis of gene expression using two-color RNA labeling in live yeast. Nature Methods. 2013;10:119-121. doi: 10.1038/nmeth.2305.

15. Larson DR, Zenklusen D, Wu B, Chao JA, Singer RH. Real-time observation of transcription initiation and elongation on an endogenous yeast gene. Science. 2011;332:475-478. doi: 10.1126/science.1202142.

16. Wang Y, Liu CL, Storey JD, Tibshirani RJ, Herschlag D, Brown PO. Precision and functional specificity in mRNA decay. Proc Natl Acad Sci USA. 2002;99:5860-5865. doi: 10.1073/pnas.092538799.

17. Siwiak M, Zielenkiewicz P, Jacobson A, Grebogi C, Kito K. A Comprehensive, Quantitative, and Genome-Wide Model of Translation. PLoS Comput Biol. 2010;6:e1000865. doi: 10.1371/journal.pcbi.1000865.

18. Belle A, Tanay A, Bitincka L, Shamir R, O'Shea EK. Quantification of protein half-lives in the budding yeast proteome. PNAS. 2006;103:13004-13009. doi: 10.1073/pnas.0605420103.

19. Ghaemmaghami S, Huh W-K, Bower K, Howson RW, Belle A, Dephoure N, et al. Global analysis of protein expression in yeast. Nature. 2003;425:737-741. doi: 10.1038/nature02046.

20. Kafri M, Metzl-Raz E, Jona G, Barkai N. The Cost of Protein Production. Cell Reports. 2016;14:22-31. doi: 10.1016/j.celrep.2015.12.015.

21. Milo R, Shen-Orr S, Itzkovitz S, Kashtan N, Chklovskii D, Alon U. Network motifs: Simple building blocks of complex networks. Science. 2002;298:824-827.

22. Sharon E, Kalma Y, Sharp A, Raveh-sadka T, Levo M, Zeevi D, et al. Articles Inferring gene regulatory logic from high-throughput measurements of thousands of systematically designed promoters. Nature Biotechnol. 2012;30:521-530. doi: 10.1038/nbt.2205.

23. Hooshangi S, Thiberge S, Weiss R. Ultrasensitivity and noise propagation in a synthetic transcriptional cascade. Proc Natl Acad Sci USA. 2005;102:3581-3586. doi: 10.1073/pnas.0408507102.

24. Benos PV, Bulyk ML, Stormo GD. Additivity in protein-DNA interactions: how good an approximation is it? Nucleic Acids Res. 2002;30:4442-4451.

25. Lee W, Tillo D, Bray N, Morse RH, Davis RW, Hughes TR, et al. A high-resolution atlas of nucleosome occupancy in yeast. Nature Genet. 2007;39:1235-44.

26. Niño CA, Hérissant L, Babour A, Dargemont C. mRNA nuclear export in yeast. Chemical Reviews. 2013;113:8523-8545. doi: 10.1021/cr400002g.

27. Smith C, Lari A, Derrer CP, Ouwehand A, Rossouw A, Huisman M, et al. In vivo single-particle imaging of nuclear mRNA export in budding yeast demonstrates an essential role for Mex67p. J Cell Biol. 2015;211:1121-1130. doi: 10.1083/jcb.201503135.

28. Mor A, Suliman S, Ben-Yishay R, Yunger S, Brody Y, Shav-Tal Y. Dynamics of single mRNP nucleocytoplasmic transport and export through the nuclear pore in living cells. Nature Cell Biol. 2010;12:543-552. doi: 10.1038/ncb2056.

29. Waldron C, Jund R, Lacroute F. Evidence for a high proportion of inactive ribosomes in slow-growing yeast cells. Biochemical Journal. 1977;168:409-415.

30. Gillespie DT. Exact stochastic simulation of coupled chemical reactions. J Phys Chem. 1977;81:2340-2361.

31. Wagner A. Energy constraints on the evolution of gene expression. Mol Biol Evol. 2005;22:1365-1374. doi: 10.1093/molbev/msi126.

32. Wagner A. Energy costs constrain the evolution of gene expression. J Exp Zool Part B. 2007;308B:322-324. doi: 10.1002/jez.b.21152.

33. Scott M, Klumpp S, Mateescu EM, Hwa T. Emergence of robust growth laws from optimal regulation of ribosome synthesis. Mol Syst Biol. 2014;10:747. doi: 10.15252/MSB.20145379.

34. Snapp EL. Fluorescent proteins: a cell biologist's user guide. Trends in Cell Biology. 2009;19:649-655. doi: 10.1016/J.TCB.2009.08.002.

35. Lynch M, Sung W, Morris K, Coffey N, Landry CR, Dopman EB, et al. A genome-wide view of the spectrum of spontaneous mutations in yeast. Proc Natl Acad Sci USA. 2008;105:9272-9277. doi: 10.1073/pnas.0803466105.

36. Roy AL, Singer DS. Core promoters in transcription: old problem, new insights. Trends Biochem Sci. 2015;40:165-171. doi: 10.1016/j.tibs.2015.01.007.

37. Cheng J, Maier KC, Avsec Ž, Rus P, Gagneur J. Cis-regulatory elements explain most of the mRNA stability variation across genes in yeast. RNA. 2017;23:1648-1659. doi: 10.1261/rna.062224.117.

38. Hinnebusch AG. Molecular Mechanism of Scanning and Start Codon Selection in Eukaryotes. Microbiology and Molecular Biology Reviews. 2011;75:434-467. doi: 10.1128/mmbr.00008-11.

39. Tuller T, Ruppin E, Kupiec M. Properties of untranslated regions of the *S. cerevisiae* genome. BMC Genomics. 2009;10:391. doi: 10.1186/1471-2164-10-391.

40. Decker KB, Hinton DM. Transcription Regulation at the Core: Similarities Among Bacterial, Archaeal, and Eukaryotic RNA Polymerases. Annu Rev Microbiol. 2013;67:113-139. doi: 10.1146/annurev-micro-092412-155756.
